# Transverse Skin Crease versus Vertical Midline Incision versus Laparoscopy for Right Hemicolectomy: A Systematic Review—Current Status of Right Hemicolectomy

**DOI:** 10.1155/2014/643685

**Published:** 2014-01-30

**Authors:** Alberto Santoro, Carlo Boselli, Claudio Renzi, Francesca Gubbiotti, Veronica Grassi, Giorgio Di Rocco, Roberto Cirocchi, Adriano Redler

**Affiliations:** ^1^Department of Surgical Sciences, “Sapienza” University of Rome, Viale Regina Elena 324, 00185 Rome, Italy; ^2^Department of General and Oncologic Surgery, University of Perugia, St. Maria Hospital, Località Sant'Andrea delle Fratte, Via Gambuli 1, 06156 Perugia, Italy; ^3^Department of General Surgery, University of Ferrara, Via Fossato di Mortara 64/A, 44121 Ferrara, Italy

## Abstract

*Purpose*. The right hemicolectomy may be conducted through laparoscopic or laparotomic surgery, transverse or midline incisions. The transverse laparotomy offers some advantages compared to the midline laparotomy and laparoscopy. A literature review was performed to evaluate the possible advantages of the transverse incision versus midline incision or laparoscopic right hemicolectomy. *Methods*. A systematic research was performed in Medline, Embase, Cochrane Central Register of Controlled Trials, CINAHL, BioMed Central, and the Science Citation Index. *Results*. Laparotomic right hemicolectomy with transverse incision is preferable to laparotomic hemicolectomy with midline incision. A transverse incision offers a lessened postoperative pain following physical activity, a lessened need to administer analgesic therapy during the post-operative time, better aesthetic results, and a better post-operative pulmonary function. Open surgery with transverse or midline incision ensured a shorter operative time, lower costs and a greater length of the incision compared to the laparoscopic. However, there are no differences in the oncological outcomes. *Conclusions*. It was not possible to identify significant differences between the open right hemicolectomy with transverse incision versus the open right hemicolectomy with midline incision or laparoscopic hemicolectomy.

## 1. Introduction

The right hemicolectomy may be conducted through laparotomic or laparoscopic (LRH) surgery, transverse or midline incisions. The open right hemicolectomy may be performed through midline incision (ORHM) or transverse/oblique incision (ORHT). According to a number of surgeons, the transverse laparotomy offers some advantages compared to the midline laparotomy, such as a less postoperative pain, a smaller alteration of the respiratory function, and better aesthetic results. Furthermore, a lower incidence of incisional hernia has been observed after an ORHT incision or LRH than after an ORHM [1–6]. For these reasons, some surgeons considered the ORHT transverse laparotomic a valid alternative to LRH in the right hemicolectomy. The objective of this systematic review is to evaluate the possible advantages of the midline or transverse incision in the right hemicolectomy for the colon cancer.

## 2. Methods

This systematic review was performed according to the Preferred Reporting Items for Systematic reviews and Meta-Analyses (PRISMA) [7] statement Eligibility criteria provided parameters of exclusion and inclusion.

### 2.1. Eligibility Criteria

#### 2.1.1. Inclusion and Exclusion Criteria

Randomized clinical trials (RCTs) and nonrandomized clinical studies (non-RCSs) which compared ORHT (open right hemicolectomy with transverse incision) versus LRH (laparoscopic right hemicolectomy), TLRH (totally laparoscopic right hemicolectomy), and/or ORHM (vertical or midline incision open right hemicolectomy), enrolling adult patients, irrespective of gender and comorbidities, were considered. No language or publication status restrictions were imposed. Studies in which the outcomes of interest were not reported or impossible to be extrapolated from the published results were excluded.

#### 2.1.2. Information Sources and Search

A systematic search was performed in Medline, Embase, Cochrane Central Register of Controlled Trials, CINAHL, BioMed Central, and the Science Citation Index for potentially relevant studies comparing laparoscopic-assisted versus vertical or open right hemicolectomy with transverse-incision for right-sided colon cancer. Search was restricted to studies published between January 1990 and December 2012. The literature search was carried out using the following medical subject headings (MeSH) and free text words: right (All Fields) AND (“colectomy” (MeSH Terms) OR “colectomy” (All Fields)) AND incision (All Fields) AND laparoscopy (All Fields). A secondary search was conducted reviewing unpublished literature databases including GreyNet, SIGLE, Current Controlled Trials, and the Cochrane Central Register of Controlled Trials. The “related articles” tool available in PubMed was used to expand the research. To minimize retrieval bias, we carried out a manual search in Google Scholar database and in 7 high-impact journals, chosen on the basis of the frequency of articles found and experts' opinion from 2010 to 2012: Diseases of the Colon & Rectum, Colorectal Disease, Archives of Surgery, British Journal of Surgery, Journal of American College of Surgery, Techniques in Coloproctology, and International Journal of Colorectal Disease.

### 2.2. Study Selection

Two authors (Roberto Cirocchi, Veronica Grassi) independently assessed titles or abstracts of all identified studies. Full-text articles of potentially relevant studies were assessed for inclusion independently in an unblended standardized manner by two authors (Francesca Gubbiotti, Claudio Renzi).

*Data Collection Process*. A data extraction sheet was developed, based on the Cochrane Consumers and Communication Review Group's data extraction template [8]. Two authors (Roberto Cirocchi, Veronica Grassi) extracted independently the data from the included studies including patients' demographics, inclusion and exclusion criteria, type of intervention, and outcomes of interest. These data were checked by a third author (Francesca Gubbiotti).

### 2.3. Statistical Analysis

On the basis of the relevant heterogeneity founded in the included studies, we consider impossible to conduct a meaningful meta-analysis of data. Consequently, we performed a descriptive, qualitative, and quantitative analysis of the outcomes of interest by summarizing retrieved data for each treatment in form of unweighted mean and range of values (min–max). Then, we assessed the number of studies that for each endpoint showed a statistical difference between the analyzed procedures (*P* < 0.05).

#### 2.3.1. Assessment of Methodological Quality of the Included Studies

The methodological quality of the included studies was assessed using the revised and modified grading system of the Scottish Intercollegiate Guidelines Network [9].

#### 2.3.2. Types of Outcome Measures

The analyzed outcomes were divided into perioperative: operative time, length of specimen, length of incision, number of lymph nodes collected, tumor size, blood loss, and transfusions; postoperative: morbidity, complications and length of hospital stay, pain, analgesia, microscopic or macroscopic infiltration of the resection margin, and reoperation rate and 30-day postoperative mortality rate.

## 3. Results

**Figure 1:**
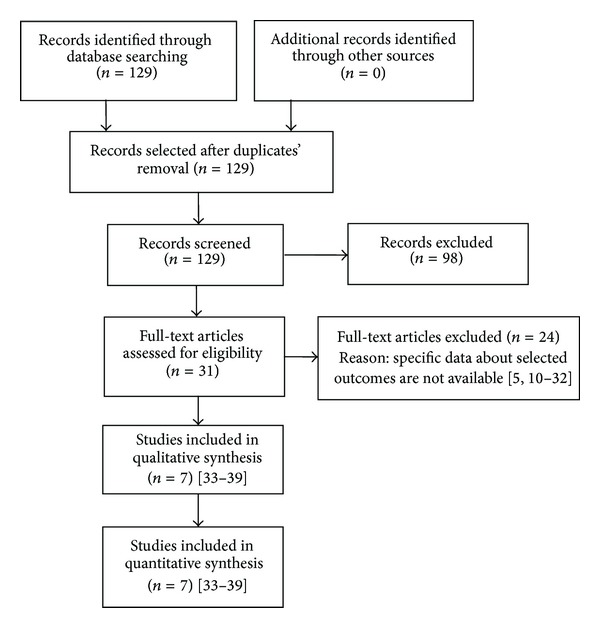
PRISMA flowchart of the literature search.

129 studies were identified by this systematic literature review; 98 studies were excluded after checking of the abstracts as they were not relevant (Figure 1). 24 further studies were excluded after evaluation of the full text in detail [5, 10–32]. Seven studies fulfilled the inclusion criteria [33–39].

### 3.1. Description of Studies

In our literature review we identified 2 RCTs [34, 35] and 5 non-RCSs studies [33, 36–39] analyzing 350 patients who underwent a right hemicolectomy (245 laparotomies, of which 141 with transverse incision and 104 with midline incision, and 105 laparoscopic interventions).

We developed a data grid compiling the characteristics of the right hemicolectomies: author, publication year, nationality of the study, period of the study, number of patients, age, sex, ASA, TNM, type of approach adopted for the right hemicolectomy (transverse or midline laparotomy, video assisted laparoscopy, and total or hand assisted laparoscopy), number of patients per approach, exclusion and inclusion criteria, type of resection, and type of anastomosis performed (Table 1).

### 3.2. Results of Methodological Quality Assessment

The methodological quality assessment performed according to the modified grading system of the Scottish Intercollegiate Guidelines Network (SIGN) [9] proved to be of fair quality for 5 of the comparative studies included. Furthermore, the study by Tanis et al. and the one by Brown et al. turned out to be both of good quality (overall mean score 13 points) (Table 2).

### 3.3. Risk of Bias in Included Studies

According to the 20 items from the revised and modified SING checklist, some methodological limits were exposed (Table 2) [9]. In particular, five studies [33, 34, 36, 38, 39] did not indicate where the authors were on the learning curve for the reported procedure (item 6). In four studies [33, 35, 37, 38] surgical technique was not standardized (item 9). None of the included studies stated the participation rate defined as the number of participants divided by the number of eligible patients (item 15). Finally, Stipa et al. and Lohsiriwat et al. [33, 36] did not perform the analysis by intention to treat (item 20). Between the included studies there was imbalance in the baseline characteristics of the participants (gender, age at surgery, BMI, pathology, TNM stage, comorbidity, ASA classification, disease location, and previous abdominal surgery) and there were differences between the kinds of compared accesses (Table 2). With regard to some outcomes, it was not possible to establish comparisons as different units of measurements were used. Some outcomes were not reported in all studies. The follow-up period for certain outcomes was not reported in some studies. In the majority of the studies the postoperative mortality is assessed at 30 days from the operation except in Stipa and Lindgren's studies where the postoperative mortality was not specified [33, 34]. The studies that reported the incisional hernia indicated different follow-up periods; for instance, Veenhof et al. indicated a follow-up period of 18 months in the LRH group and of 20 months in the open group [38]. As for the overall postoperative morbidity only Veenhof and Tanis' studies specified that the follow-up period was of 30 days [38, 39].

Overall, since some outcomes have been evaluated in different ways, the results cannot be statistically compared. Furthermore because of the type of studies included (2 RCTs and 5 non-RCTs) the risk of bias assessed was mild-moderate.

### 3.4. Effects of Interventions

#### 3.4.1. Postoperative Morbidity, Wound Infection, and Incisional Hernia

Only in Veenhof and Tanis' studies it was specified that the follow-up period for postoperative morbility was of thirty days [38, 39]. Five studies reported the wound infections [33, 34, 36–38]; only Veenhof and Tanis' studies evaluated incisional hernia (Table 3).

*ORHT versus ORHM*. Overall post-operative morbidity that was bigger in the ORHM group (41% versus 20%) [39]. Stipa et al. reported a greater number of wound infections in the ORHM group [33]. In Tanis' study a median followup of 44 months was performed; a higher incision of incisional hernia was observed in the ORHM group compared to the ORHT group [39].

*ORH versus LRH*. Tan et al. reported a bigger number of post-operative complications in the LRH group but notstatistically significant [37].

*ORHM versus LRH*. Tanis et al. reported a bigger number of post-operative complications in the ORHM group compared to the LRH group (41% versus 20%) [39]. Lohsiriwat's study reported a higher number of wound infections in the ORHT group [36].

*ORHT versus LRH*. Lohsiriwat et al. reported a bigger overall post-operative morbidity in the ORHT group (1/20 versus 0/13) [36]; conversely, Tanis et al. reported a bigger overall postoperative morbidity in the LRH group (20% versus 13%) [39]. In Tanis study a higher incidence of incisional hernia was highlighted in the Assisted-Lap group [39].

*ORHT versus TLRH*. Veenhof et al. reported a bigger number of post-operative complications, in particular wound infections, in the ORHT group compared to the TLRH group [38].

### 3.5. Length of PostOperative Hospital Stay

Three studies reported the length of post-operative stay [33, 37, 39]. However, different units of measurement were used. Four studies mentioned the total hospital stay [34, 36–39] (Table 4).

*ORHM versus ORHT.* No significant difference was reported [33, 34, 39].

*ORH versus LRH*. Tan et al. did not report any significant difference in the length of the hospital stay [37].

*ORHM versus LRH*. Length of the post-operative hospital stay was higher in the ORHM group compared to the LRH group (8 days versus 6 days) [39].

*ORHT versus LRH*. No statistically significant differences were reported [36, 39].

### 3.6. Operative Time

All the included studies reported this outcome, but data were not comparable as different units of measurements were used (Table 4).

*ORHM versus ORHT*. Stipa and Tanis' studies indicated a slightly longer operative time in the ORHM group but not in a statistically significant manner [33, 39], while Brown's study reported a greater duration in the ORTH group [35]. There were not statistically significant differences between the two groups.

*ORH versus LRH*. Operative time was shorter in the ORH compared to LRH group [37].

*ORHM versus LRH*. In Tanis's study a less operative time in the ORHM group was reported; this difference was statistically significant [39].

*ORHT versus LRH*. In Lohsiriwat's study a statistically significant shorter operative time was observed in the ORHT group [36].

*ORHT versus TLRH*. The operative time was shorter in the ORHT group (*P* = 0.001) [38].

### 3.7. Length of Specimen and Tumor Size

Only two studies [38, 39] reported the length of the specimen. Different units of measurements were used (Table 5).

*ORHT versus ORHM*. The length of the specimen was bigger in the ORHM group compared with the ORHT and Assisted-Lap groups [39].

*ORHT versus TLRH*. In Veenhof's study the length of the specimen was significantly greater in the laparoscopic group, but not statistically significant. The neoplasia was bigger in the ORHT group than in the Total-Lap group (*P* = 0.13) [38].

*ORHT versus LRH*. The length of the specimen was greater in the ORHT group but in a nonstatistically significant manner [36, 39].

*ORHM versus LRH*. In Tanis's study the length of the specimen was significantly greater in the ORHM compared with the Assisted-Lap group [39].

*ORH versus LRH*. Both the length and the diameter of the neoplasia were bigger in the open group compared with the Assisted-Lap group, but these differences were not statistically significant [37].

### 3.8. Number of Harvested Lymph Nodes

It was not possible to compare this outcome, as different units of measurement were used (Table 5).

*ORHT versus ORHM*. Brown reported a higher, but not statistically significant, number of removed lymph nodes in the ORTH group compared with the ORHM group [35].

*ORH versus LRH*. In Tan's study the number of harvested lymph nodes (HL) was bigger in the LRH group compared with the open group but nonstatistically significant.

*ORHM versus LRH*. Tanis's study reported a bigger number of HL in the Assisted-Lap group compared with the ORHM group (*P* = 0.63) [39].

*ORHT versus LRH*. Tanis and Lohsiriwat's studies reported a bigger number of removed lymph nodes in the Assisted-Lap group compared with the ORHT group, but the outcome was not statistically relevant [36, 39].

*ORHT versus TLRH*. In Veenhof's study the number of removed lymph nodes was bigger in the Total-Lap group compared with the ORHT group (*P* = 0.49) [38].

### 3.9. Intraoperative Blood Loss and Transfusion

Only Lohsiriwat et al. and Veenhof et al. reported the intraoperative blood loss [36, 38]. Unfortunately, it was not possible to compare this outcome as different units of measurement were used: the mean ± SD [36] and the median and interquartile range [38]. Only two studies [37, 38] (Veenhof and Tan) reported the number of transfusions performed.

*ORH versus LRH*. In Tan's study the number of patients having received a blood transfusion peri- and postoperatively was bigger in the open group (median and transverse incision) (8/40) than in the Assisted-Lap (20% versus 14%), but this difference was not statistically significant (*P* = 0.549) [37].

*ORHT versus LRH*. In Lohsiriwat's study the intraoperative blood loss was smaller in the open group compared to the Assisted-lap group (107, 5 ± 40, 6 mL versus 120, 8 ± 57, 9 mL (mean ± standard deviation)), but in a nonstatistically significant manner (*P* = 0.48) [36].

*ORHT versus TLRH*. In Veenhof's study the intra-operative blood loss was greater in the ORHT group compared to the Total-Lap group (130 mL versus 60 mL (median interquartile range)); this difference was statistically relevant (*P* = 0.001) [38]. As regards the transfusion there were no statistically significant differences between the two groups; indeed, no patient required a blood transfusion as a direct consequence of the operation [38].

### 3.10. Postoperative Pain and Postoperative Analgesia

Only two studies [33, 34] referred to the intensity of pain. Lindgren et al. evaluated the pain intensity both after rest and after physical activity (standing up beside the bed, coughing, moving around, etc.) using the pain core (VAS) scale [34]. Stipa et al. classified the intensity of the post-operative pain on the basis of the quantity of analgesics required by the patient [33]. Five studies reported the type and length of the post-operative analgesic therapy [34–37, 39]. However, data were not comparable because different units of measurement were used, the post-operative pain was evaluated according to different parameters, and analgesic therapy administered varied between the studies (Table 6).

*ORHT versus ORHM*. The intensity of pain after physical activity, in the first three days following the operation, was more severe in the ORHM group [34]. In Stipa's study a higher percentage of patients reported post-operative pain of medium-moderate grade in the ORHM group compared with the ORHT group; similarly, a higher percentage of patients reported post-operative pain of moderate-severe grade in the ORHT group compared with the ORHM group [33]. In Brown's study the median total dose of morphine used in the ORHT group was greater [35]. Instead in Lindgren's study, a bigger dose of analgesics was administered to the ORHM [34].

*ORH versus LRH*. Tan et al. did not report significant differences between the two groups as for the length of the parenteral analgesic therapy (2 days) [37].

*ORHM versus LRH*. Tanis et al. reported a greater length of the analgesic therapy 3 days after ORHM compared with the LRH group [39].

*ORHT versus LRH*. The length of the discontinued analgesic therapy administered through parental route that was bigger in the ORHT group than in the Assisted-Lap group [36].

### 3.11. Microscopic (R1) or Macroscopic Infiltration of the Resection Margin (R2)

Only Brown et al. and Lohsiriwat et al. reported an explicit statement of microscopic (R1) or macroscopic (R2) infiltration of the resection margin [35, 36]. In Veenhof's study the radical resection was reported while Tanis' study reported the positive resection margin [38].

No statistically significant differences were reported between the four types of treatment.

### 3.12. Length of Incision

Four studies [34–37] reported the length of the incision. In Lohsiriwat's study it was not specified whether the length of the incision, in the Assisted-Lap group, was the length of the incision for the extraction of the specimen or the addition of the length of the incisions [36]. On the contrary, in Tan's study the length of the incision mentioned for the laparoscopic group was the incision to extract the specimen and it did not include the cumulative length of all the trocar incisions [37]. It was not possible to compare this outcome as the units of measurements used in the included studies were different (Table 7).

*ORHM versus ORHT*. In Brown's study the length of the incision was minor in the ORTH group compared to the ORHM group, statistically significant. [35].

*ORH versus LRH*. Tan et al. reported a length of the incision smaller in the Assisted-Lap group compared to the ORH group either with transverse incision or with median incision (*P* < 0.01) [37].

*ORHT versus LR*. In Lohsiriwat's study the length media of the incision was bigger in the ORTH group compared with the Assisted-Lap group in a statistically significant manner (*P* < 0.001) [36].

### 3.13. Time to First Bowel Movement

Veenhof's study was the only one that did not report the recovery time after the first bowel movement [38]. However, it was not possible to compare this outcome as different units of measurement were used in the included studies (Table 8).

*ORHM versus ORHT*. The first bowel movement was slightly faster in the ORTH group [34, 35].

*ORH versus LRH*. No statistically significant differences were reported [37].

*ORHM versus LRH*. A statistically significant faster resumption of the bowel movement in the Assisted-Lap group was reported [39].

*ORHT versus LRH*. In Lohsiriwat's study a faster resumption of the bowel movement in the Assisted-Lap group was reported [36].

### 3.14. Time to Resumption of Normal Diet

Brown's study reported both the time for resumption of a liquid diet and the time required for resuming the solid diet [35]. However, it was not possible to compare this outcome as different units of measurements were used in the included studies (Table 8).

*ORHM versus ORHT*. Brown's study reported a faster time, nonstatistically significant, to resumption of liquid diet in the ORHT group [35]. The study of Lindgren reported a faster time to resumption of normal diet in the ORHT group, nonstatistically significant [34].

*ORH versus LRH*. No statistically significant differences were reported [37].

*ORHM versus LRH*. Tanis et al. did not report statistically significant differences [39].

*ORHT versus LRH*. In Lohsiriwat's study the Assisted-lap group showed a shorter time to resumption of normal diet compared to the ORHT group [36].

### 3.15. Time to Defecation

It was not possible to compare this outcome as the included studies used different units of measurement [33, 34, 36] (Table 8).

*ORHM versus ORHT*. In Stipa's study the time to defecation in the first four days from the operation was observed in a higher percentage of patients in the ORHT group compared with the ORHM group (53% versus 46%), not statistically significant [33].

*ORHT versus LRH*. time to defecation was shorter in the Assisted-lap group (*P* = 0.25) [36].

### 3.16. Cost

Only Veenhof et al. reported the costs of the operation that were significantly bigger in the Assisted-lap group compared to the ORHT group (7221€ versus 6033€; *P* = 0.03) [38].

### 3.17. Pulmonary Function

Only Lindgren study reported this outcome and compared between the ORHT and the ORHM groups [34]. The oxygen saturation in the first day after the operation was similar in the two groups ORHM 95, 1 ± 0.4 versus ORHT 95, 1 ± 0.8. In both groups a fall in the FEV1 (forced expiratory volume I second) was observed after the operation; this fall was higher and longer in the ORHM group compared to the ORHT (*P* < 0.05). With regard to the CV (vital capacity), its fall was higher and longer in the ORHM group versus the ORHT (*P* < 0.05) [34].

### 3.18. Reoperation

In Lohsiriwat and Tanis' studies it was specified that the reoperation was carried out within 30 days from the operation [36, 39].

*ORHM versus ORHT*. In Tanis' study a greater number of patients in the ORHM group required a reoperation within 30 days because of serious complications: 1 band dehiscence; 2 anastomic leaks; 1 intestinal perforation (4/22 versus 0/23) [39].

*ORH versus LRH*. No differences between the two groups and no reoperations were reported [37].

*ORHT versus LRH*. Lohsiriwat and Tanis' studies did not report any differences [36, 39].

*ORHT versus TLR*. In Veenhof's study 2 patients of the Total-Lap group had to undergo a reoperation due to an anastomotic leak in one case and a prolonged ileum in the other case [38].

### 3.19. Postoperative Mortality

In all studies the post-operative mortality at 30 days was mentioned except for the studies of Stipa and Lindgren that show the post-operative mortality without specifying which one was evaluated [33, 34] (Table 9). As regards this outcome no statistically significant difference between the four different surgical approaches was detectable.

## 4. Discussion

Presently it is controversial whether the transverse incision offers advantages compared with the median incision and the laparoscopy. Langer's line of cleavage crosses the skin of the anterior abdominal wall in a transverse direction. An incision parallel to these lines will therefore cause the least structural and cosmetic damage. A vertical incision therefore divides the fascial fibers of the anterior abdominal wall, that lie in a transverse direction, and suture closure of such vertical wound places the suture material between the fibers. Contraction of the abdominal wall causes laterally directed tension on the closure line and might cause the suture material to cut through by separation of the transversely orientated fibers [40, 41]. From our literature review it was not possible to prove that the right hemicolectomy with transverse incision laparotomy presents significant advantages when compared to the open right hemicolectomy with midline incision or with the laparoscopic right hemicolectomy; this is due to the small number of studies and the high heterogeneity of the data reported. A few comparative studies have elaborated outcomes that show statistically significant differences, such as operative time, intraoperative blood loss, postoperative pain, post-operative analgesia, length of the incision, time to first bowel movement, costs, and pulmonary function. On the contrary, no statistically significant differences were found between the three groups with regard to postoperative morbidity, occurrence of incisional hernia, wound infection, length of post-operative hospital stay, length of the specimen, number of harvested lymph nodes, tumor size, transfusions, infiltration of the resection margins, time to defecation and time to flatus, time to resumption of normal diet, reoperations, post-operative mortality. Concerning the comparison between the open surgery and the laparoscopic surgery, statistically significant differences were observed with regard to the operative time (which was shorter in the open surgery [37]) and to the length of the incision (which was shorter in the laparoscopic surgery [37]). The advantages of the ORHT compared with the ORHM consist in a less post-operative pain after physical activity, a diminished need to administer analgesic therapy during the post-operative time [34], a shortened length of the incision [35], and a better post-operative pulmonary function [34]. Comparing the laparoscopic right hemicolectomy to the open hemicolectomy with transverse incision, the advantages of the laparoscopy are a shortened length of the incision [36] and a decreased blood loss [38]. With respect to the open right hemicolectomy with transverse incision, its advantages are a shorter operating time [36] and a lower cost [38]. Lastly, when comparing the laparoscopic right hemicolectomy to the open right hemicolectomy with median incision, the laparoscopy offers the advantage of a quicker recovery of peristalsis [39] while the open right hemicolectomy with median incision ensures a less operative time [39]. From our review of the literature it is shown that the ORHT is preferable to the ORHM as it offers a lessened post-operative pain following physical activity, a lessened need to administer analgesic therapy during the post-operative time, better aesthetic results, and a better post-operative pulmonary function; however, there are no differences about the oncologic outcomes; these data are confirmed by other studies in the literature. Indeed, the Cochrane review of Brown and Goodfellow [42], comparing the transverse incision with the midline incision in the upper and lower abdominal surgery, demonstrates that the transverse and oblique incisions have a weaker impact on the pulmonary function, especially in the early post-operative days, while a reduced tendency to dehiscence and infection of the surgical wound and to the appearance of the incisional hernia is observed; furthermore, the transverse incision appears to be associated with less pain, although the data concerning this outcome are rather scarce and unclear. The authors of this review conclude that the differences in the outcomes between the two types of incisions are minimal, so the choice depends on the surgeon's preference. The midline incision is preferable in emergency as it ensures a more rapid access to the abdominal cavity in patients who have a high risk of relaparotomy or in those where it is expected the packaging of an anastomosis. The transverse incision may be preferred in obese patients or in patients with reduced pulmonary function [42]. In his review, Grantcharov and Rosenberg reached similar conclusions, stating that the transverse incision seems preferable to the median on the basis of anatomical and physiological principles ensuring less complications in the early post-operative period and a reduced incidence of hernia of incision [1] In a similar manner, our review shows that only in some studies a few statistically significant differences emerge in terms of outcomes; these differences are not such as to determine a clear preference between the two types of incision. With regard to the comparison between laparoscopic and open access, the open access offers the advantage of a lower operative time in spite of a greater length of the incision; also in this case, there are no differences in some of the outcomes such as mortality or morbidity for which it is not possible to state the superiority of one of the two accesses. These data are confirmed by the literature that does not show significant differences about the oncologic outcome [18, 20, 37, 43, 44]. Some studies suggest that laparoscopy has advantages compared to open surgery in terms of less post-operative hospital stay, quicker recovery of peristalsis, less post-operative pain [18–20], lower incidence of surgical infections [44], best aesthetic results compared to higher cost [37]. As regards the comparison between open access with transverse incision and the laparoscopic access, our review found that the benefits of open access with transverse incision are represented by lower operative time and lower costs while the benefits of laparoscopy are the shorter length of incision and less blood loss. Consequently, currently the choice of one of these accesses is entrusted by the experience and personal preference of the surgeon, as in the literature there are no data showing any real benefits of the ORHT compared to the ORHM or LRH.

## 5. Conclusion

Our study compares the open right hemicolectomy with transverse incision with the midline incision and laparoscopic hemicolectomy; the studies chosen were a mixed bag ranging from small RCT to retrospective studies creating a rather heterogeneous sample. Based on their data, there were no real significant differences. Currently, in the setting of minimally invasive surgery (robotic, laparoscopy, and SILS), the advantages of a transverse skin crease laparotomy are not clear. This approach is not easy and is often limited by colon anatomy, BMI, and size of lesion. Why would anyone choose this approach over conventional laparoscopy which provides better visualization and potentially less trauma to the lesion. Currently the choice of the surgical access is up to the surgeon, on the basis of his experience and preference as well as of the patient's characteristics. With a view to highlighting the most significant differences among the three groups, high-powered randomized clinical trials would be required.

## Figures and Tables

**Table 1:** Characteristics of the included studies.

Author/year/design/setting	Time lapse	Age	Gender	Number of patients			Resection	Anastomosis
				ORH^1^	TLRH^2^	LRH^3^		
Stipa et al., 2000 [33] CCT^4^ Rome Italy	1998 1999	ORHM^5^ 63 ORHT^6^ 69	NA^7^	(i) 17 ORHT Incision started 1 cm above the umbilicus to the anterior axillary line (ii) 27 ORHM (60% xiphopubic, 40% xiphoumbilical)			NA	NA

Lindgren et al., 2001 [34] RCT^8^ Goteborg Sweden	2000	NA	NA	(i) 23 ORHM (ii) 17 ORHT Incision from the left hypochondrium rostral to the umbilicus to the right flank dividing the right half of the rectus muscle ± part of the left rectus			NA	NA

Brown Et Al., 2004 [35] Rct Sheffield UK	Aug 2001 Aug 2002	ORHM 70 (30–90) ORHT 74 (42–86)	ORHM F 6 (43%) ORHT F 8 (57%)	(i) 14 ORHT Incision following the skin crease from the midline 1 cm above the umbilicus (ii) 14 ORHM			NA	NA

Lohsiriwat et al., 2007 [36] CCT Bangkok Thailand	2004 2006	LRH 56.9 ± 13.5 ORH 65.2 ± 6.0	LRH F 53.8 ORH F 65.O	20 ORHT Incision on the right side of abdomen about 1 cm above the umbilicus		13	Extracorporeally	Side-to-side stapled ileocolic anastomosis was performed extracorporeally

Tan et al., 2009 [37] CCT Singapore Singapore	2005 2007	LRH: M 19 (51%) F 18 (49%) ORH: M22 (55%) F 18 (45%)	LRH 68 (37–83) ORH 67 (42–87)	(i) 18 (45%) Midline incision (ii) 22 (55%) Right transverse skin crease incision on the right flank		(i) 29 Transverse (78%) (ii) 8 Vertical (22%)	The intracorporeal ligature of vascular pedicle was performed with either laparoscopic linear staples or with LigaSure vessel sealing system. Resection of bowel was performed extracorporeally	End-to-end ileocolic anastomosis was performed extracorporeally with linear staples

Veenhof et al., 2010 [38] CCT Amsterdam Holland The Netherlands	2005 2009	LRH 68 (61–79) ORHT 75 (67.78)	LRH M 13 (52%) F 12 (48%) ORHT M 9 (32%) F 19 (68%)	28 ORHT Incision on the right side of abdomen about 1 cm above the umbilicus	25		NA	NA

Tanis et al., 2012 [39] CCT Amsterdam The Netherlands	2006 2009	LRH 75 (31–85) ORHM 74 (47–83) ORHT 73 (54–85)	LRH 12 (40%) ORHM M 9 (41%) ORHT M 10 (43%)	(i) 23 ORHT (ii) 22 ORHM		30	NA	Ileocolic anastomosis was performed extracorporeally

^1^Open right hemicolectomy.

^2^Totally laparoscopic right hemicolectomy.

^3^Laparoscopic-assisted right hemicolectomy.

^4^Clinical controlled trial.

^5^Open right hemicolectomy median incision.

^6^Open right hemicolectomy transverse incision.

^7^Not available.

^8^Randomized controlled trial.

**Table 2:** Evaluation of methodological quality of the included studies.

Items/author*	Stipa et al., 2000 [33]	Lindgren et al., 2001 [34]	Brown et al., 2004 [35]	Lohsiriwat et al., 2007 [36]	Tan et al., 2009 [37]	Veenhof et al., 2011 [38]	Tanis et al., 2012 [39]
Inclusion criteria	1	1	1	1	1	1	1
Exclusion criteria	0	1	1	1	1	1	1
Comparable demographics?	1	1	1	1	1	1	1
Could the number of participating centers be determined?	1	1	1	1	1	1	1
Could the number of surgeons who participated be determined?	0	0	1	1	0	0	1
Could the reader determine where the authors were on the learning curve for the reported procedure?	0	0	1	0	1	0	0
Were diagnostic criteria clearly stated for clinical outcomes if required?	1	1	1	1	1	1	1
Was the surgical technique adequately described?	1	1	1	1	1	1	1
Did they try to standardize the surgical technique?	0	1	0	1	0	0	1
Did they try to standardize perioperative care?	0	1	1	0	1	0	1
Were the age and range given for patients in the ORHT group?	1	0	1	1	0	1	1
Did the authors address whether there were any missing data?	0	1	1	0	0	1	1
Were the age and range given for patients in the comparative group(s)?	1	0	1	1	1	1	1
Were patients in each group treated along similar timelines?	1	1	1	1	1	1	1
The patients asking to enter the study, did they actually take part in it?	0	0	0	0	0	0	0
Were dropout rates stated?	0	1	0	0	0	0	0
Were outcomes clearly defined?	1	1	1	1	0	1	1
Were there blind assessors?	0	0	1	0	0	0	0
Were there standardized assessment tools?	0	1	1	0	0	0	0
Was the analysis by intention to treat?	0	1	1	0	1	1	1

Score	9	14	17	12	11	12	15

Total score: 20; <8: poor quality; 8–14: fair quality; ≥15: good quality.

*Named by reference number and listed by publication date.

**Table 3:** Postoperative (30 days) morbidity, wound infection, and incisional hernia.

Study	Postoperative complications	Surgical technique			
		ORHM^1^	ORHT^2^	LRH^3^	TLRH^4^
Stipa et al. [33]	Complications/patient (%)	7/27 (25)	1/17 (6)		
	Wound infection	3	1		
	Pleural effusion	1	0		
	DVT^5^	1	0		
	Hemorrhage	1	0		
	UTI^6^	1	0		
	Incisional hernia	NR^7^	NR		

Lindgren et al. [34]	Complications/patient (%)	8/23 (35)	2/17 (12)		
	Wound infection	1	0		
	Pneumonia	3	0		
	Anastomotic bleeding	1	0		
	Subcutaneous wound rupture	1	0		
	Bowel obstruction	2	0		
	Atrial fibrillation	0	1		
	UTI	0	1		
	Incisional hernia	NR	NR		

Brown et al. [35]	Complications/patients (%)	2/14 (14)	3/14 (21)		
	Wound infection	NR	NR		
	Prolonged ileus	1	2		
	Chest infection	0	1		
	Rectal bleeding 1/14	1	0		
	Incisional hernia	NR	NR		

Lohsiriwat et al. [36]	Wound infection/patients (%)		1/20 (5)	0	
	Incisional hernia		NR	NR	

Tan et al. [37]	Complications/patients (%)	ORH (ORHM + ORHT) 2/40 (5)		5/37 (14)	
	Wound infection	1		2	
	Intra-abdominal abscess	0		1	
	Cardiac complication	1		1	
	Respiratory complication	0		1	
	Incisional hernia	NR		NR	

Veenhof et al. [38]	Postoperative complications/patient (%)		9/28 (32)		7/25 (28)
	Wound infection		2		1
	Pneumonia		2		2
	Anastomotic leak		0		1
	Ileus		4		3
	UTI		4		1
	Incisional hernia		3		1

Tanis et al. [39]	Postoperative complications/patient (%)	10/22 (41)	3/23 (13)	6/30 (20)	
	Wound infection	NR	NR	NR	
	Incisional hernia	2	0	2	

^1^Open right hemicolectomy median incision.

^2^Open right hemicolectomy transverse incision.

^3^Laparoscopic-assisted right hemicolectomy.

^4^Totally laparoscopic right hemicolectomy.

^5^Deep venous thrombosis.

^6^Urinary tract infection.

^7^Not reported.

**Table 4:** Length of post-operative hospital stay (POS) (days) and operative time (OT) (minutes).

Study	Outcome (*p*)	ORHM ^1^	ORHT^2^	LRH^3^	TLRH^4^
Stipa et al. [33]	POS	≤8: 2/28 (7)^5^ ≥8: 26/28 (93)	≤8: 8/17 (47) ≥8: 9/17 (53)		
	OT	157^6^ 155 (120–200)^7^	107 110 (80–120)		

Lindgren et al. [34]	POS	8.9 ± 0.6^8^	7.5 ± 0.7		
	OT	135.6 ± 7.5^9^	135.7 ± 7.4		

Brown et al. [35]	POS	7 (3–16)^7^	7 (4–14)		
	OT	51 (41–100)^10^	61 (45/105)		

Lohsiriwat et al. [36]	POS		7.1 ± 2.6^10^	6.2 ± 2.4	
	OT (<0.001)		105 ± 24^11^	208 ± 57	

Tan et al. [37]	POS	ORH^12^ 5^7^		5	
	OT	ORH^12^ 72 (35–160)^6^		111 (65–190)	

Veenhof et al. [38]	POS		9 (6–12)^13^		8 (6–12)
	OT (0.001)		77 (62–88)^13^		155 (115–184)

Tanis et al. [39]	POS	8 (3–55)^7^	7 (4–24)	6 (3–23)	
	OT: ORHM versus LRH (0.001)	105 (63–173)^7^	101 (65–173)	129 (85–177)	

^1^ Open right hemicolectomy median incision.

^2^Open right hemicolectomy transverse incision.

^3^LRH laparoscopic assisted right hemicolectomy.

^4^TLRH totally laparoscopic right hemicolectomy.

^5^ *n* Patients/Tot patients (%).

^6^Mean (range).

^7^Median (range).

^8^Total hospital stay; Mean ± SEM (SEM: standard error of the mean).

^9^Mean ± SEM (standard error of the mean).

^10^Mean ± 2 SD (standard deviation).

^11^Mean ± SD (standard deviation).

^12^ORH: open right hemicolectomy.

^13^Median (IQR: interquartile range).

**Table 5:** Length of the specimen (LS), tumor size (TS) (cm), and number of harvested lymph nodes (*N*).

Study	Outcome (*p*)	ORHM^1^	ORHT^2^	LRH^3^	TLRH^4^
Stipa et al. [33]	LS; TS	NR^5^	NR		
	*N*	NR	NR		

Lindgren et al. [34]	LS; TS	NR	NR		
	*N*	NR	NR		

Brown et al. [35]	LS & TS	NR	NR		
	*N* (NS)	10 (3–21)^6^	11 (6–19)		

Lohsiriwat et al. [36]	TS		6.1 ± 2.6^7^	5.7 ± 2.7	
	*N*		18.8 ± 10.8 (7–47)^7^	29.2 ± 18.1 (5−66)	

Tan et al. [37]	TS: ORH^8^ versus LRH (0.06)	ORH: *Ø* 4.35^9^; TS length 4.35^9^		Ø 3.9; TS length 4.2	
	*N*: ORH versus LRH (0.174)	ORH 15^9^		18	

Veenhof et al. [38]	LS (0.09)		LS6 22 (17–26)^10^		LS 26 (22–32)
	TS (0.13)		TS 4 (3.4–5)^10^		TS 5 (3.3–6)
	*N* (0.49)		14 (8–19)^10^		15 (12–19)

Tanis et al. [39]	LS: ORHM versus LRH (0.06)	LS 26 (17–44)^6^	LS 25 (19–43)	LS 22 (8–40)	
	*N*: ORHM versus LRH (0.63)	13.5 (2–38)^6^	13 (5–36)	15 (1–28)	

^1^Open right hemicolectomy median incision.

^2^Open right hemicolectomy transverse incision.

^3^LRH laparoscopic assisted right hemicolectomy.

^4^TLRH totally laparoscopic right hemicolectomy.

^5^Not reported.

^6^Median (range).

^7^Mean ± SD (standard deviation).

^8^Open right hemicolectomy.

^9^Mean.

^10^Median (IQR: interquartile range).

**Table 6:** Postoperative pain and analgesia.

Study	Parameter/therapy details	ORHM^1^	ORHT^2^	LRH^3^	TLRH^4^
Stipa et al. [33]	Pain intensity: visual analogic scale	No-mild 12/17 (70%)	No-mild 11/27 (40%)		
		Moderate-severe 5/17 (30%)	Moderate-severe 17/27 (60%)		

Lindgren et al. [34] ORHM versus ORHT *P* < 0.05	Average total amount ± SD analgesics given	85 ± 9.8 mg	50 ± 7.9 mg		

Brown et al. [35]	Median doses of morphine (range)	94 mg (21–565)	101 mg (59–219)		
	Discontinued patient-controlled analgesia (days)	3 (2–7)^5^	4 (2–5)		

Lohsiriwat et al. [36] ORHT versus LRH *P* = 0.25	Time (days) to discontinuation of IV^6^ narcotics		1.4 ± 1.0^7^	1.0 ± 0.9	

Tan et al. [37] ORH versus LRH *P* = 0.478	Median time of narcotic usage (days)	ORH^8^ 2		2	

Veenhof et al. [38]			NR^9^		NR

Tanis et al. [39] ORHM versus ORHT *P* = 0.430	Stop to parenteral analgesia (days): epidural (91%) or patient-controlled (9%) equally distributed between groups	3 (1–6)^5^	2 (1–6)		

^1^Open right hemicolectomy median incision.

^2^Open right hemicolectomy transverse incision.

^3^LRH laparoscopic assisted right hemicolectomy.

^4^TLRH totally laparoscopic right hemicolectomy.

^5^Median (range).

^6^Intravenous.

^7^Mean ± 2 standard deviation.

^8^Open right hemicolectomy.

^9^Not reported.

**Table 7:** Length of incision cm.

Study	ORHM^1^	ORHT^2^	LRH^3^	TLRH^4^
Stipa et al. [33]	NR^5^	NR		
Lindgren et al. [34]	18 (12–24)^6^	18 (12–13)		
Brown et al. [35]	11 (10–19)^6^	10 (7–15)		
Lohsiriwat et al. [36]		10.3^7^	7.7	
Tan et al. [37]	11.2 (6–12)^6^		5.6 (3–10)	
Veenhof et al. [38]		NR		NR
Tanis et al. [39]	NR	NR	NR	

^1^Open right hemicolectomy median incision.

^2^Open right hemicolectomy transverse incision.

^3^LRH laparoscopic assisted right hemicolectomy.

^4^TLRH totally laparoscopic right hemicolectomy.

^5^Not reported.

^6^Mean (range).

^7^Median.

**Table 8:** Time to first bowel movement (Bowel), time to resumption of normal diet (Diet), and time to defecation (Def) (days).

Study	Outcomes (days) (*p*)	ORHM^1^	ORHT^2^	LRH^3^	TLRH^4^
Stipa et al. [33]	Bowel	NR^5^	NR		
	Diet	NR	NR		
	Def	≤4: 13 (46%)^6^ >4: 15 (54%)	≤4: 9 (53%) >4: 8 (47%)		

Lindgren et al. [34]	Bowel	18 (12–24)^7^	18 (12–13)		
	Diet	4.3 ± 0.5^8^	3.5 ± 0.3		
	Def	NR	NR		

Brown et al. [35]	Bowel	11 (10–19)^7^	10 (7–15)		
	Diet	Oral fluids intake 3 (1–9)^9^	2 (1–9)		
		Solid diet 4 (2–10)^9^	4 (2–10)		
	Def	7 (3–16)^9^	7 (4–14)		

Lohsiriwat et al. [36]	Bowel		10.3^9^	7.7	
	Diet (0.39)		4.3 ± 1.1^10^	3.9 ± 1.0	
	Def (0.25)		3.7 ± 1.8^10^		3.3 ± 0.9

Tan et al. [37]	Bowel (0.23)	ORH^11^: 11.2 (6–12)^7^		5.6 (3–10)	
	Diet (0.33)	ORH: 2 days^9^		2 days	
	Def	NR		NR	

Veenhof et al. [38]	Bowel		NR		NR
	Diet		NR		NR
	Def		NR		NR

Tanis et al. [39]	Bowel	NR	NR	NR	
	Diet ORHM versus LRH (0.62)	3 (1–9)^9^	3 (1–10)	3 (1–17)	
	Def	NR	NR	NR	

^1^Open right hemicolectomy median incision.

^2^Open right hemicolectomy transverse incision.

^3^LRH laparoscopic-assisted right hemicolectomy.

^4^TLRH totally laparoscopic right hemicolectomy.

^5^Not reported.

^6^Days: *n* patients (%).

^7^Mean (range).

^8^Mean ± SEM.

^9^Median (range).

^10^Mean ± 2 SD.

^11^Open right hemicolectomy.

**Table 9:** Post-operative mortality (*n* patients (%)).

Study	ORHM^1^	ORHT^2^	LRH^3^	TLRH^4^
Stipa et al. [33]	0/27	0/17		
Lindgren et al. [34]	0/23	0/17		
Brown et al. [35]	0/14	0/14		
Lohsiriwat et al. [36]		0/20	0/13	
Tan et al. [37]	0/18	0/22	0/37	
Veenhof et al. [38]		1/28 (4%)		1/25 (4%)
Tanis et al. [39]	0/22	0/23	0/30	

^1^Open right hemicolectomy median incision.

^2^Open right hemicolectomy transverse incision.

^3^Laparoscopic assisted right hemicolectomy.

^4^Totally laparoscopic right hemicolectomy.
